# Contrast-enhanced echocardiographic diagnosis of benign and malignant cardiac tumors and its correlation with pathology

**DOI:** 10.3389/fcvm.2023.1182334

**Published:** 2023-06-08

**Authors:** Zihao Yang, Yicui Niu, Hui Ma, Wenqing Gong, Lu Yu, Liwen Liu, Minjuan Zheng

**Affiliations:** ^1^ Department of Ultrasound, Xijing Hospital, The Fourth Military Medical University, Xi’an, China; ^2^ Department of Pathology, Xijing Hospital, The Fourth Military Medical University, Xi’an, China

**Keywords:** echocardiography, contrast-enhanced echocardiography, cardiac tumors, differential diagnosis, immunohistochemistry, correlation analysis

## Abstract

**Background:**

This study aimed to explore the diagnostic value of contrast-enhanced echocardiography (CEE) in benign and malignant cardiac tumors and detect the correlation of CEE parameters and immunohistochemistry (IHC) markers.

**Methods:**

The data of 44 patients with cardiac tumors confirmed by pathology were reviewed. Lesions were examined before surgery using transthoracic echocardiography (TTE) and CEE with time-intensity curve analysis. The expression of CD31, VEGF and Ki67 was measured by IHC staining. Microvessel density (MVD) was quantified via IHC for CD31. The clinical variables, TTE, CEE and IHC parameters were compared between benign and malignant cardiac tumors. Receiver operating characteristic curve were used to analyze the value of factors in predicting malignant cardiac tumors. The correlation between CEE and IHC parameters was analyzed.

**Results:**

Among 44 cardiac tumors, 34 were benign and 10 were malignant. There were significant differences in the TTE parameters (pericardial effusion, tumor boundary, diameter, basal width), CEE parameters (tumor peak intensity (TPI), peak intensity ratio of tumor to myocardium (TPI/MPI), area under time-intensity curve (AUTIC)) and IHC parameters (Ki67, MVD, CD31, VEGF) between the benign and malignant tumor groups (all *P *< 0.05). Receiver operating characteristic curve analysis showed that the CEE and IHC parameters had diagnostic value in malignant cardiac tumors. There was a correlation between TPI/MPI and Ki67 (*r* = 0.62), AUTIC and Ki67 (*r* = 0.50), and AUTIC and CD31 (*r* = 0.56).

**Conclusion:**

TTE and CEE parameters were different between benign and malignant cardiac tumors. CEE is helpful to differentiate the properties of cardiac tumors. There is a correlation between CEE parameters and IHC markers. AUTIC and TPI/MPI can reflect the proliferation and invasion of tumors.

## 1. Introduction

According to the World Health Organization's classification, cardiac tumors are divided into benign, malignant and intermediate tumors of uncertain behavior, and there are over 35 types of primary cardiac tumors in total (1). The incidence rate of primary cardiac tumors is approximately 0.001%–0.28%, as reported by a large autopsy series (2). A study reported that approximately 18% of tumor patients in the progressive stage suffered cardiac metastases (3). The therapy and prognosis is based on the nature and clinical stage of the cardiac tumor (3). Generally, benign tumors have a favorable prognosis after surgical resection, while the prognosis of malignant tumors is poor (4).

Cardiac tumors are polymorphic and complex, and although computed tomography and magnetic resonance imaging can provide qualitative diagnosis in a few types of cardiac tumors (such as myxoma, lipoma and cardiac fibroma), preoperative imaging diagnosis is still a challenge (5). As a first-line clinical examination method, echocardiography plays an important role in the evaluation of cardiac space-occupying lesions. Contrast-enhanced echocardiography (CEE) can help distinguish thrombi from tumors and evaluate the blood supply of masses (6, 7). Thus, the aims of this study were to further compare the perfusion difference between benign and malignant cardiac tumors and to explore the correlation between CEE parameters and immunohistochemistry (IHC) markers of proliferation and vascularization, which could provide a reference for the treatment and prognosis of cardiac tumor patients.

## 2. Materials and methods

### 2.1 Study subjects

From October 2015 to September 2021, a total of 44 cardiac tumor patients in Xijing Hospital who underwent surgery and were confirmed by pathology were enrolled in this study (18 males and 26 females, mean age 52.6 ± 16.8 years, range 21–76 years). Each patient underwent transthoracic echocardiography (TTE) and CEE, and 32 patients also underwent computed tomography and magnetic resonance imaging. This study was approved by the ethics committee of Xijing Hospital, The Fourth Military Medical University (KY20162034−1).

### 2.2. Conventional and contrast-enhanced echocardiography

TTE was performed using a Philips EPIQ7C ultrasound system with an S5−1 transducer (Philips Ultrasound Inc), and the transmission frequency was 1.7–3.4 MHz. Patients were placed in the left lateral decubitus position, and electrocardiogram (ECG) was recorded. Abnormal ECG including T wave or ST segment depression, atrial or ventricular premature beats, and bundle branch block. Conventional standard TTE was performed in all 44 patients according to the guidelines of the Chinese Society of Echocardiography (8). In addition, the location, size, boundary, echogenicity, and mobility of the cardiac masses (texture of tumor was soft or hard and could be reflected by its movement with heart beating) were specifically observed. Whether the boundary between the tumor margin and surrounding normal cardiac tissue was clear was also assessed by echocardiography.

Sonovue (Bracco Inc, Milan, Italy) was used as the ultrasound contrast agent in this study. The left ventricular opacification mode with a low mechanical index (0.1–0.3) was selected. One milliliter of SonoVue suspension was intravenously injected slowly, followed by 5 ml saline injection (9). The instant flash technique (mechanical index 1.0–1.5) was used to observe the blood perfusion process in the mass and adjacent or middle interventricular septum myocardium. The steps above were repeated three times for each subject. All the image loops (each loop at least long for 15 cardiac cycles) were stored for further offline analysis.

CEE data were analyzed by the image analysis software QLAB 13.0 (Philips Ultrasound Inc). The region of interest was tracked within the tumors and the adjacent or middle interventricular septum myocardium. The region of interest box was set as 5 mm × 5 mm and placed in the maximum enhancement area inside the tumor. The motion compensation function of the software was used to ensure that the region of interest box was inside the target. When the time-intensity curve was calculated, the peak intensity of the tumors (TPI) and of the myocardium (MPI) which adjacent the tumors or middle interventricular septum (when tumors located in pericardium, right atrium or great vessel), area under time-intensity curve (AUTIC), slope of perfusion curve and time to peak (TPK) were measured. All measurements were performed by two experienced sonographers blinded to the surgery and pathology results.

### 2.3. Pathology

Formalin-fixed specimens were paraffin-embedded and cut into 4-µm-thick sections. IHC staining was evaluated with the classic streptavidin-peroxidase method, with CD31 for observation of blood vessels, with VEGF for evaluation of new blood vessel formation and with Ki67 for evaluation of proliferation. All specimens were evaluated with the following markers: rabbit anti-Ki67 (1:100, #GB111141, Servicebio, Wuhan, China), rabbit anti-VEGF (1:100, #GB13034, Servicebio, Wuhan, China), and rabbit anti-CD31 (1:100, #GB11063−1, Servicebio, Wuhan, China). All markers were used to perform immunostaining together with biotin-labeled secondary antibodies (1:200, #GB23303, Servicebio, Wuhan, China) and a DAB (3,3’diaminobenzidine) detection system, followed by hematoxylin counterstaining. DAB binds to proteins marked immunohistochemically, causing it to appear brown, and stained cells were counted as positive. Negative controls were obtained by omitting the primary antibody.

Morphometric analysis was performed using Image-Pro Plus (Version 4.0; Media Cybernetics, USA). Identification and counting of tumor vessels and positive cells were determined independently by two pathologists blinded to the clinical characteristics. The results of Ki67, CD31 and VEGF were calculated by the mean ratio of positive cells to all cells within each section per tumor of three random fields at the 400 high-power fields (10). Microvascular density (MVD) was quantified by counting the number of vessels plus CD31 immunoreactive positive endothelial cells in three vascular “hot spots” within the tumor at 200 high-power fields (the result expressed as the mean NO. of vessels/field) (11).

### 2.4. Statistical analyses

SPSS (version 23.0; SPSS Inc., Chicago, IL, USA) was used for statistical analysis. The numerical data conforming to the normal distribution were expressed as the mean ± standard deviation, and independent sample *t-*tests were used for comparison. The numerical data that did not meet the normal distribution were expressed as median (interquartile range), and Mann‒Whitney *U* tests were used for comparison. The categorical data were expressed as percentages and evaluated by the Person Chi-square test (*χ^2^*). Receiver operating characteristic (ROC) curve were established to evaluate the predictive ability of potential indicators for CEE and IHC. Significance for all statistical comparisons was set at a *P-*value* *< 0.05.

## 3. Results

### 3.1. Pathological results and location of cardiac tumors

According to the final pathological results (Table 1), there were 34 benign and 10 malignant cardiac tumors. Myxoma accounted for the largest proportion of all cases (21/44). In these myxoma cases, 61.90% (13/21) were located in the left atrium, and 95.24% (20/21) were connected with the endocardium by the pedicle.

**Table 1:** Pathology and location of cardiac tumors (n = 44).

Histologic type	*n* (%)	Location
**Benign**	34 (77.27)^a^	
Myxoma	21 (61.76)^b^	LA (13), LV (2), RA (5), Bilateral (1)
Papillary fibroelastoma	4 (11.76)^b^	RV (1), PA (1), LV (2)
Lipoma	3 (8.82)^b^	Bilateral (1), RA (1), RV (1)
Leiomyoma	3 (8.82)^b^	IVS + RV + RA (1), RA + IVC (2)
Hemangioma	2 (5.88)^b^	RV (1), LV (1)
Cardiac fibroma	1 (2.94)^b^	RA (1)
**Malignant**	10 (22.73)^a^	
Endocardial sarcoma	2 (20.00)^c^	PA (1), LV (1)
Myxoid liposarcoma	1 (10.00)^c^	Pericardium (1)
Undifferentiated cardiac sarcoma	1 (10.00)^c^	LV (1)
Osteosarcoma	1 (10.00)^c^	LA (1)
Hepatic cellular cancer	1 (10.00)^c^	Pericardium + LA (1)
Adrenal undifferentiated sarcoma	1 (10.00)^c^	Bilateral + Pericardium + IVC (1)
Classical Hodgkin's lymphoma	1 (10.00)^c^	Pericardium (1)
Lung cancer	1 (10.00)^c^	Bilateral + PA (1)
Seminoma	1 (10.00)^c^	Pericardium (1)

LA, left atrium; LV, left ventricle; RA, right atrium; RV, right ventricle; IVC, inferior vena cava; PA, pulmonary artery.

^a^ Among total patients studied, i.e., out of 44.

^b^ Among benign tumors, i.e., out of 34.

^c^ Among malignant tumors, i.e., out of 10.

### 3.2. Comparison of clinical, TTE, CEE and IHC between benign and malignant cardiac tumors

**Figure 1:**
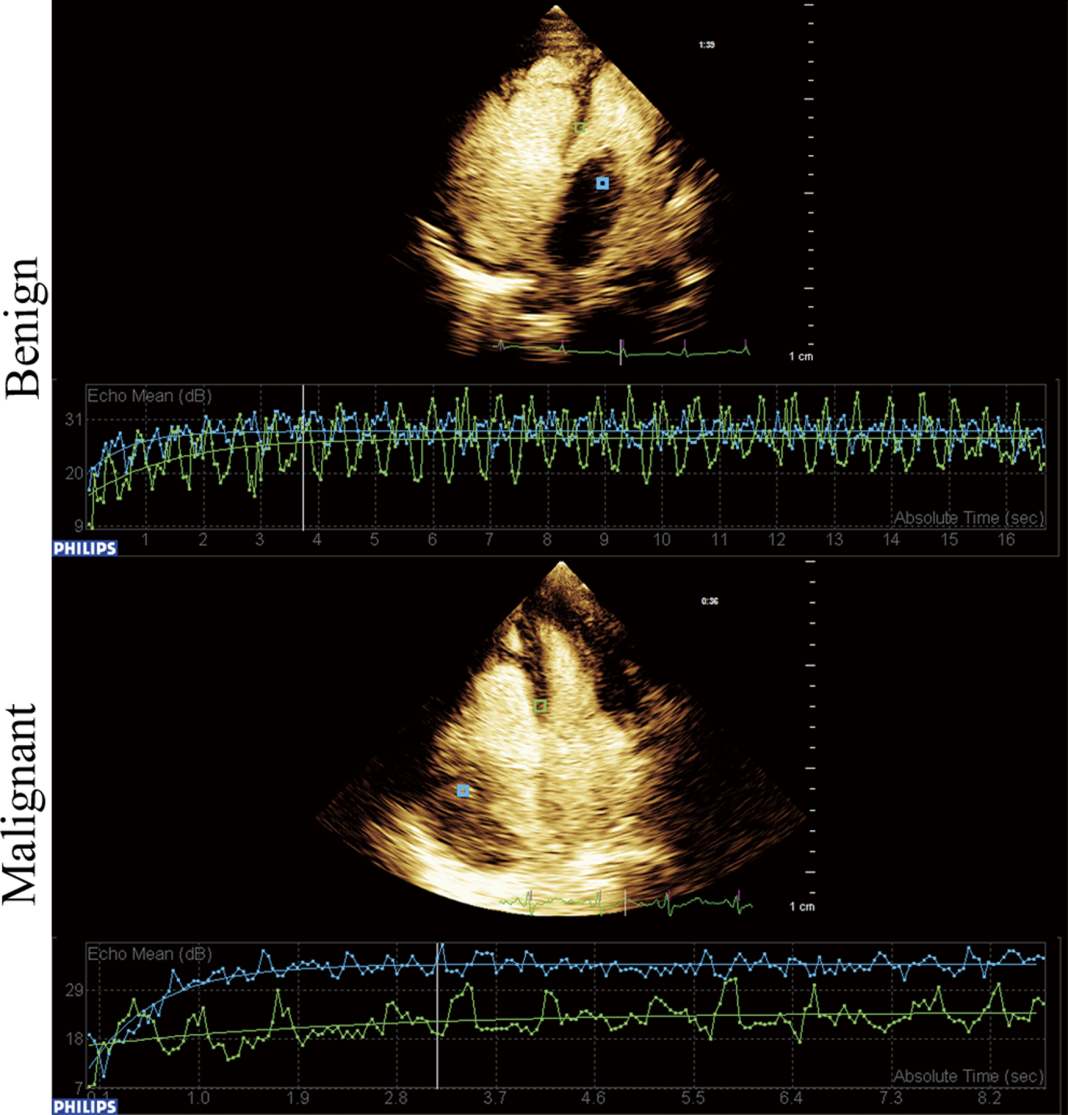
Time-intensity curve of benign and malignant cardiac tumors. Blue frame located in the tumor and green frame located in the myocardium.

**Figure 2:**
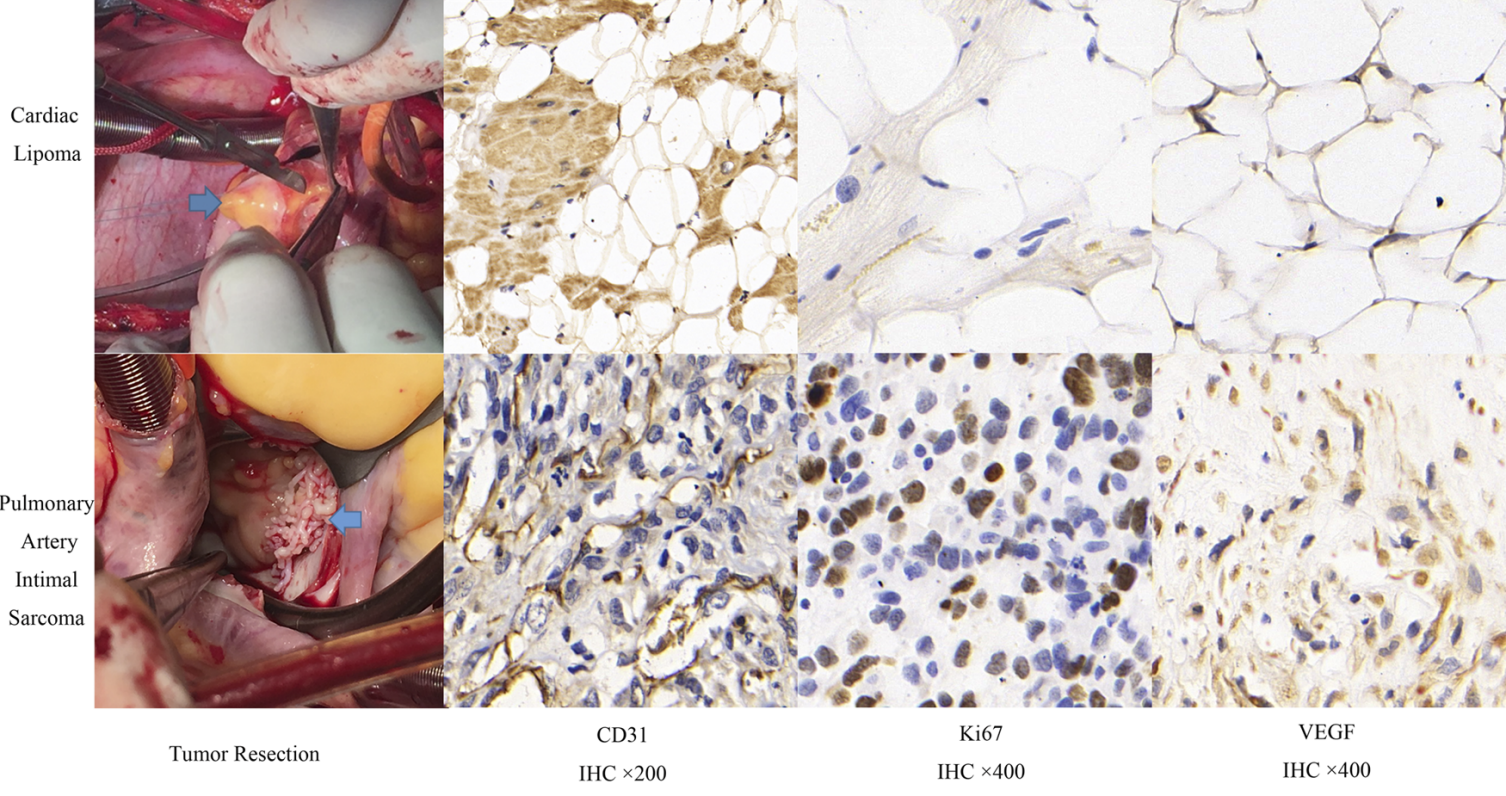
Comparison of surgical resection and immunohistochemistry of benign and malignant cardiac tumors.

The comparison of clinical symptoms (including murmur, palpitation, chest distress and angina), TTE, CEE and IHC variables are shown in Table 2. Twenty-two (50%) patients showed abnormal ECG, including T wave or ST segment depression (17 cases), atrial or ventricular premature beats (2 cases), and bundle branch block (5 cases). Three patients had 2–3 types of abnormal ECG manifestations simultaneously. Tumor diameter, basal width, unclear boundary, and pericardial effusion were significantly different between benign and malignant tumors (*P *< 0.05). Quantitation of TPI, TPI/MPI, AUTIC, MVD, CD31, VEGF and Ki67 in the benign group was lower than that in the malignant group (*P *< 0.05). Taking the myocardium as a reference, the intensity of ultrasound contrast agent by malignant tumors is usually greater than that of benign tumors (Figure 1). Benign tumors generally have a regular shape, complete encapsulation, and rare hemorrhage and necrosis, while malignant tumors generally manifest as highly pleomorphic and poorly differentiated cells (Figure 2).

**Table 2:** Comparison of clinical, echocardiography and histology variables between benign and malignant cardiac tumors.

Variables	Benign (*n* = 34)	Malignancy (*n* = 10)	*x^2^/U/t*	*P–*value
Age (years)	51.50^a^	52.50^a^	151.000	0.594
Male, *n* (%)	14 (41.18)	4 (40.00)	0	1
Clinical symptoms, *n* (%)	22 (64.71)	5 (50.00)	0.072	0.788
Abnormal electrocardiogram, *n* (%)	15 (44.11)	7 (70.00)	0.001	0.982
Diameter (mm)	19.29^a^	33.40^a^	279.000	0.002
Basal width (mm)	19.53^a^	32.60^a^	271.000	0.004
Irregular shape, *n* (%)	21 (61.76)	8 (80.00)	0.476	0.490
Unclear boundary, *n* (%)	8 (23.53)	8 (80.00)	8.348	0.004
Tumor mobility, *n* (%)	18 (52.94)	5 (50.00)	0	1
Hyperechoic texture, *n* (%)	25 (73.53)	5 (50.00)	1.037	0.309
Obstruction, *n* (%)	15 (44.11)	6 (60.00)	0	1
Pericardial effusion, *n* (%)	4 (11.76)	5 (50.00)	4.792	0.029
LVEF (%)	58.58 ± 3.77^b^	57.00 ± 3.97^b^	1.100	0.278
TPI (dB)	18.77^a^	25.10^a^	72.000	0.006
TPI/MPI	0.79^a^	1.49^a^	52.000	0.001
AUTIC (dB sec)	75.32 ± 37.97^b^	129.29 ± 42.11^b^	−3.15	0.004
Slope (dB/sec)	4.01^a^	7.49^a^	68.000	0.374
TPK (sec)	6.63 ± 2.62^b^	6.89 ± 1.71^b^	−0.244	0.809
MVD (NO. of vessels/field)	1.67^a^	13.67^a^	69.000	0.005
Ki67 (%)	1.90^a^	33.70^a^	8.000	<0.001
CD31 (%)	4.80^a^	12.30^a^	50.000	0.001
VEGF (%)	8.90^a^	30.70^a^	58.000	0.002

TPI, tumor peak intensity; MPI, myocardium peak intensity; AUTIC, area under time-intensity curve; TPK, time to peak; MVD, Microvessel density.

^a^ The values are presented as medians.

^b^ The values are presented as the mean ± standard deviation.

### 3.3. Diagnostic values of TTE, CEE and IHC parameters in differentiating malignant cardiac tumors

**Figure 3:**
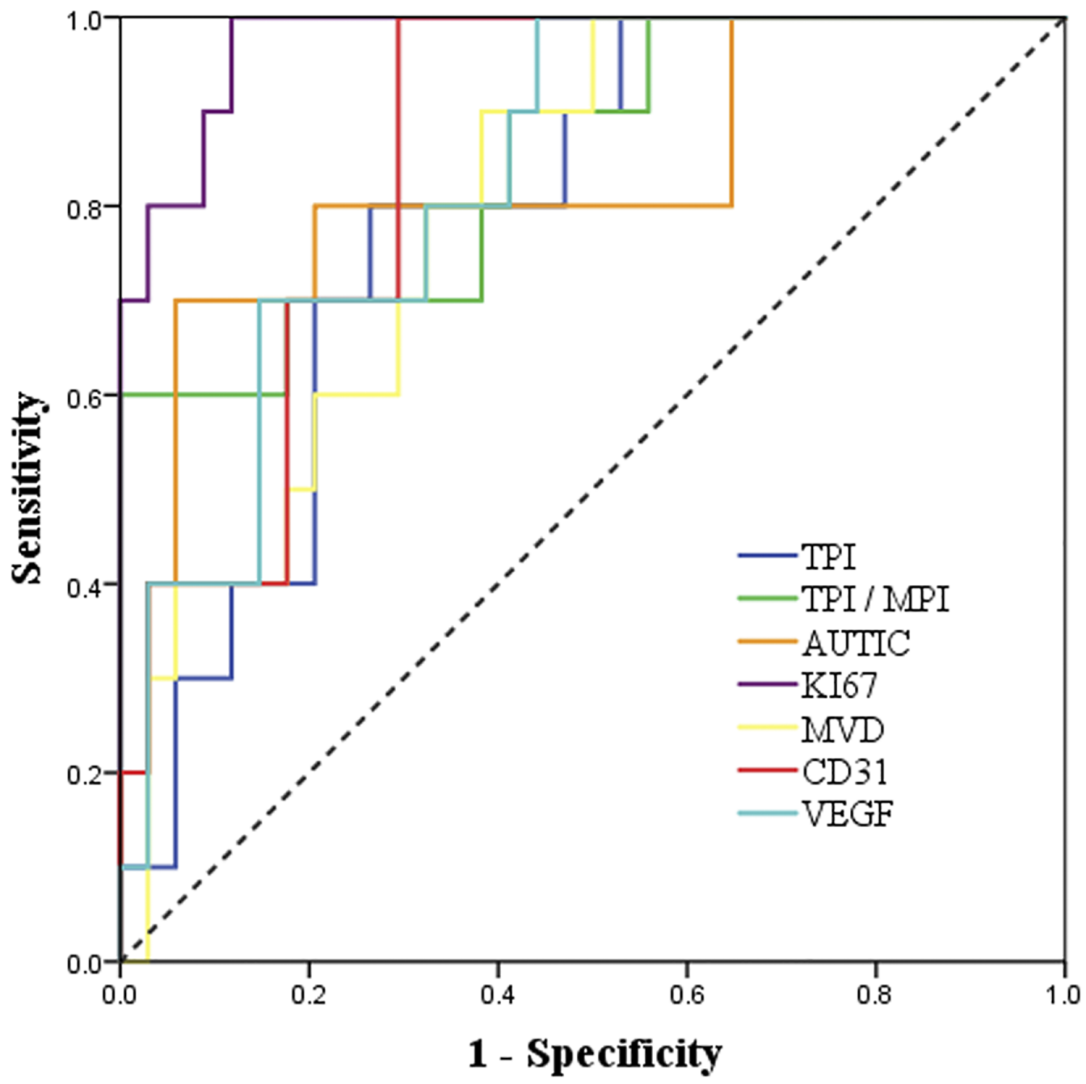
ROC curve analysis of the diagnostic value of CEE and IHC parameters for malignant cardiac tumors.

Based on the final pathological results, we analyzed the diagnostic value of TTE, CEE and IHC parameters for predicting benign and malignant tumors. The diameter, basal width, unclear boundary, pericardial effusion, TPI, TPI/MPI, AUTIC and all IHC parameters had high diagnostic values (Table 3 and Figure 3).

**Table 3:** CEE and IHC parameters in differentiating malignant cardiac tumors.

Variables	AUC (95% CI)	cutoff point	sensitivity	specificity	*P-*value
Diameter	0.77 (0.63, 0.92)	31.50	100.00%	50.00%	0.010
Basal width	0.82 (0.66, 0.98)	33.50	60.00%	91.20%	0.002
Unclear boundary	0.73 (0.55, 0.92)	0.73	70.00%	76.50%	0.027
Pericardial effusion	0.61 (0.40, 0.82)	0.61	40.00%	82.40%	0.287
TPI	0.79 (0.65, 0.93)	22.97	80.00%	73.50%	0.006
TPI/MPI	0.85 (0.71, 0.99)	1.34	60.00%	100.00%	0.001
AUTIC	0.83 (0.66, 0.99)	125.06	70.00%	94.10%	0.002
MVD	0.80 (0.66, 0.93)	3.00	90.00%	61.80%	0.005
Ki67	0.98 (0.94, 1)	13.20%	100.00%	88.20%	<0.001
CD31	0.85 (0.74, 0.97)	7.15%	100.00%	70.60%	0.001
VEGF	0.83 (0.70, 0.96)	12.50%	100.00%	55.90%	0.002

AUC, the area under the ROC curve; CI, confidence interval; TPI, tumor peak intensity; MPI, myocardium peak intensity; AUTIC, area under the time-intensity curve; MVD, microvessel density.

### 3.4. Correlation between CEE and IHC parameters

The correlation analysis between CEE and IHC parameters is shown in Table 4. There was a correlation between TPI/MPI and Ki67, AUTIC and Ki67, and AUTIC and CD31. No significant correlation existed among the other parameters.

**Table 4:** Correlation analysis between CEE and IHC parameters.

Variables	TPI		TPI/MPI		AUTIC	
	*r*	*P-*value	*r*	*P-*value	*r*	*P-*value
MVD	0.34	0.024	0.22	0.144	0.13	0.403
Ki67	0.37	0.014	0.62	<0.001	0.50	<0.001
CD31	0.03	0.833	0.19	0.220	0.56	<0.001
VEGF	0.06	0.684	0.14	0.370	0.35	0.021

*r*, correlation coefficient; TPI, tumor peak intensity; MPI, myocardium peak intensity; AUTIC, area under the time-intensity curve; MVD, microvessel density.

## 4. Discussion

The pathological types of cardiac tumors are complicated. Primary cardiac tumors arise from various parts of the heart, such as fatty tissue, endocardium, myocardium and nerve tissue (1). Almost all malignant tumors may metastasize to the heart (12, 13). Currently, the preoperative diagnosis of cardiac tumors mainly relies on multimodal imaging. As there are many types of cardiac tumors and their morphology is complex, the location of the tumor is variable, so generally speaking, it is difficult to determine the tumor type only by 2D echocardiography except for myxoma (5, 14). In this research, we studied parameters from TTE, CEE and corresponding pathological markers of cardiac tumors, and these immunohistochemical indicators (ki67, CD31, VEGF) are related to proliferative mobility, blood perfusion and vascularization of cardiac tumors.

In this study, 75.0% of the patients with primary malignant tumors had abnormal electrocardiograms, which may be related to primary malignant tumors infiltrating the cardiac conduction system and compressing the cardiovascular system (4). When we observed the tumor by TTE, we found that the texture, shape, mobility and obstruction of different tumors had distinct manifestations, which were mainly related to the location and size of the tumor rather than its nature, which is consistent with a previous investigation (15). By exploring various variables between benign and malignant cardiac tumors, we found some imaging indicators suggest malignant tumors. TTE showed that the diameter, basal width and unclear boundary of the tumors and pericardial effusion had statistical significance in differentiating benign and malignant cardiac tumors (*P *< 0.05), which is related to the rapid growth and invasion of malignant tumors, which need abundant blood supply (16).

When analyzing CEE data, we used TPI/MPI (peak intensity ratio of tumor to myocardium) to reduce the difference in ultrasound contrast agent uptake among different individuals. TPI, TPI/MPI and AUTIC, which represent the microcirculation perfusion of tumors (17), showed significant differences in benign and malignant tumors. However, there was no difference in TPK and slope value between the benign and malignant groups, the two CEE parameters represent tumor blood perfusion velocity and may be related to the increase in neovascularization in malignant tumors (18). Necrosis may occur inside some large and fast-growing malignant tumors without sufficient blood supply, which affects the peak intensity measurement (19). In this study, three cases of malignant tumors showing TPI less than MPI were fast-growing types: undifferentiated cardiac sarcoma, endometrial sarcoma and osteosarcoma. Of note is myxoma because the uptake of ultrasound contrast agent by myxoma was different. In 21 cases of myxoma, the highest TPI was 38.60 dB, and the lowest was 7.30 dB, while the highest and the lowest TPI/MPI were 1.21 and 0.25, respectively, which may be related to the uncertainty of biomolecular features of some myxomas (20). It has been reported that some myxoma cells overproduce CXC chemokines and interleukins, showing malignant features and metastasis (21). There has been a report of using doxorubicin and ifosfamide to prevent the recurrence of myxoma after resection (22).

To the best of our knowledge, this is the first study on the diagnostic value of CEE and its correlation with cardiac tumor pathology. As a cell proliferation marker, Ki67 expression is closely related to tumor growth, invasion and metastasis (23). VEGF is a cytokine expressed in vascular endothelial cells to promote angiogenesis (24). CD31 participates in cell adhesion and regulates angiogenesis, it can be used to estimate the number of MVDs and as a “gold standard” reflecting the number of neovascularizations (25). The clinical significance of MVD, Ki67, CD31 and VEGF has been confirmed in the diagnosis of a variety of tumors (26–28). In this study, these variables were significantly lower in the benign group than in the malignant group (*P *< 0.05). Similar to CEE, the individual differences in Ki67, VEGF, MVD and CD31 in myxoma were significant. For VEGF quantification, the lowest case was 2.5%, and the highest case was 96.4%. The expression of these IHC markers in cardiac tumors was related to their properties. For example, the expression of VEGF was low in papillary fibroelastoma and lipoma without neovascularization (0%–7.0%), while the lowest malignant tumor was 14.1% (osteosarcoma), and the highest malignant tumor was 94.4% (undifferentiated sarcoma of adrenal gland).

In differentiating benign and malignant tumors, the highest specificity was TPI/MPI and was up to 100%, and the sensitivity was only 60%, which may be related to the rich blood supply of some benign tumors (29). Compared with CEE, the sensitivity of IHC parameters was higher, in which Ki67 showed great clinical significance (sensitivity = 100%, specificity = 88.2%, AUC = 0.98, *P *< 0.001). However, the specificity of VEGF is low, which may be related to the high expression of VEGF in some benign tumors, such as hemangioma. There was a certain correlation between CEE and IHC parameters, among which the correlation of TPI/MPI and Ki67 was the strongest (*r* = 0.62). In addition, AUTIC is also correlated with Ki67 and CD31. There was no significant correlation between other parameters. High expression of Ki67 and CD31 presented rapid growth and angiogenesis of tumors, and it was usually correlated with more abundant capillaries, which is consistent with an increase in AUC (30, 31). In addition, the ultrasound contrast agent failed to show early dysfunctional microvessels in tumor tissue (32), which may explain why there is the lack of an obvious correlation between MVD and CEE parameters.

## 5. Conclusion and limitations

Our study identified that CEE has important value in the differential diagnosis of cardiac tumors. TPI/MPI and AUTIC show correlations with Ki67 and CD31, which can reflect the proliferative ability of tumor cells and vascular endothelial cells in tumors. Our results can provide a reference for the preoperative diagnosis of the degree of malignancy of cardiac tumors.

The main limitations are as follows: First, this study was performed in a single center. Second, cardiac tumors are rare with low morbidity, and we only evaluated biopsy- or surgery-proven masses. There are also many highly suggestive patients with cardiac tumors that cannot be included due to the lack of surgical or biopsy results, which leads to the study sample being limited, especially malignant tumors. Finally, due to tumor mobility with heartbeat, systematic error in the quantitative analysis of CEE may exist in different individuals, and the degree of vascularization and microcirculation perfusion of cardiac tumors need to be further studied.

## Data Availability

The raw data supporting the conclusions of this article will be made available by the authors, without undue reservation.
